# Small-molecule Bcl-2 inhibitors sensitise tumour cells to immune-mediated destruction

**DOI:** 10.1038/sj.bjc.6603599

**Published:** 2007-02-20

**Authors:** J D Lickliter, J Cox, J McCarron, N R Martinez, C W Schmidt, H Lin, M Nieda, A J Nicol

**Affiliations:** 1 Clive Berghofer Cancer Research Centre, Queensland Institute of Medical Research, Herston, Queensland 4029, Australia; 2 Department of Medical Oncology, Royal Brisbane and Women's Hospital, Herston, Queensland 4029, Australia; 3 School of Medicine, Yokohama City University, Yokohama, Japan; 4 University of Queensland Centre for Immune and Targeted Therapy, Greenslopes Private Hospital, Greenslopes, Queensland 4120, Australia

**Keywords:** Bcl-2 inhibitor, cancer immunotherapy, ABT-737, HA14-1, NKT cells

## Abstract

The cytotoxic effects of anticancer immune cells are mediated by perforin/granzyme-B, Fas ligand and tumour necrosis factor-related apoptosis-inducing ligand (TRAIL), and therefore depend on intact apoptotic responses in target tumour cells. As killing by all three of these mechanisms is blocked by the frequently overexpressed antiapoptotic oncoprotein Bcl-2, we hypothesised that coexposure to a Bcl-2 inhibitor might enhance anticancer immune responses. We evaluated this in U937 lymphoma cells, and A02 melanoma cells, which both show strong Bcl-2 expression. V*α*24^+^ V*β*11^+^ natural killer T (NKT) cells expanded from peripheral blood of normal donors (*n*=3) were coincubated with PKH26-labelled U937 cells, and cytotoxicity was determined by flow cytometry after annexin-V-FITC and 7-AAD staining. In all cases, addition of the HA14-1 small-molecule Bcl-2 inhibitor to the cocultures significantly increased apoptosis in the target U937 cells. Using a similar assay, killing of A02 cells by the cytotoxic T-lymphocyte clone 1H3 was shown to be amplified by coexposure to the potent small-molecule Bcl-2 inhibitor ABT-737. Experiments with immune effectors preincubated with concanamycin-A suggested that sensitisation to perforin/granzyme-B may underlie enhanced target-cell killing observed in the presence of Bcl-2 inhibitors. We conclude that immune destruction of malignant cells can be amplified by molecular interventions that overcome Bcl-2-mediated resistance to apoptosis.

Anticancer agents such as radiation therapy and chemotherapy kill tumour cells largely by inducing programmed cell death or apoptosis. This is also true for anticancer immune responses, where apoptosis is triggered by the mechanisms including perforin/granzyme-B, Fas ligand (FasL) and tumour necrosis factor-related apoptosis-inducing ligand (TRAIL). However, tumours frequently overexpress genes that inhibit apoptosis and which therefore contribute to treatment resistance (Cory *et al*, 1999). The Bcl-2 family of antiapoptosis mitochondrial proteins (which also includes Bcl-x_L_ and Mcl-1) is an important example of this. These proteins are potent inhibitors of cell death, and high expression levels are observed in many cancers (Adams and Cory, 1998; Reed, 1999). Bcl-2 itself is highly expressed in the majority of cases of acute leukaemia, approximately 50% of diffuse large B-cell lymphomas and up to 90% of malignant melanomas (Banker *et al*, 1997; Jansen *et al*, 1998; Campos *et al*, 1999; Skinnider *et al*, 1999). Forced expression of Bcl-2 results in resistance to a variety of proapoptotic stimuli, including growth factor withdrawal, cytotoxic chemotherapy and radiation, and there is some evidence that Bcl-2 also promotes resistance to anticancer immune responses. For example, transfection-mediated overexpression of Bcl-2 rendered Jurkat leukaemia cells refractory to killing by a cytotoxic T-cell clone (Torigoe *et al*, 1994). In other studies, enforced Bcl-2 expression was shown to inhibit several key mechanisms of immune killing, including those initiated by granzyme-B, FasL and TRAIL (Pinkoski *et al*, 2001; Raisova *et al*, 2001; Fulda *et al*, 2002; Guseva *et al*, 2002; Velthuis *et al*, 2002).

Interfering with the expression or function of Bcl-2 might be an effective anticancer strategy. Antisense oligonucleotides that decrease Bcl-2 expression caused direct tumour suppression and increased sensitivity to cytotoxic chemotherapy in preclinical models (Cotter *et al*, 1994; Jansen *et al*, 1998). However, Bcl-2 antisense therapy had limited activity when tested in clinical trials, potentially owing to poor access to the intracellular compartment and inadequate reduction in Bcl-2 expression levels (Waters *et al*, 2000; Yuen and Sikic, 2000; Morris *et al*, 2002). More recently, small molecules that target the BH3-domain-binding site of Bcl-2 have been developed as Bcl-2 inhibitors (Wang *et al*, 2000; Oltersdorf *et al*, 2005). It is hypothesised that these agents displace proapoptotic BH3-only proteins (e.g., Bid and Bim) from their binding groove on Bcl-2, thus promoting interaction with Bax and Bak, and transduction of an apoptotic signal (Oltersdorf *et al*, 2005). One of these compounds – ethyl 2-amino-6-bromo-4-(1-cyano-2-ethoxy-2-oxoethyl)-4H-chromene-3-carboxylate (designated HA14-1) – directly triggered apoptosis in Bcl-2-expressing leukaemia and lymphoma cell lines, and also enhanced the cytotoxicity of the antileukaemia drug cytarabine (Wang *et al*, 2000; Lickliter *et al*, 2003). A more-recently developed compound, ABT-737, bound to Bcl-2 and Bcl-x_L_ with extremely high affinity (inhibitory constant *K*_i_⩽1 nM), and exhibited significant *in vitro* and *in vivo* activity in preclinical models of follicular lymphoma and small-cell lung cancer (Oltersdorf *et al*, 2005). In addition, ABT-737-induced apoptosis in primary patient-derived samples of follicular lymphoma cells and chronic lymphocytic leukaemia cells.

Inhibition of Bcl-2 may be of particular benefit in combination with other strategies that promote apoptosis of malignant cells. As Bcl-2 overexpression may protect malignant cells from antitumour immune responses, we investigated whether coexposure to both Bcl-2 inhibitor and activated immune cells could increase tumour-cell killing. This hypothesis was examined in two models of anticancer immunotherapy. The first utilised human V*α*24^+^ V*β*11^+^ T-cell receptor (TCR) expressing natural killer T cells (NKT cells) as the immune effector cells (Nieda *et al*, 1999). Natural killer T cells have been shown to exert significant *in vitro* and *in vivo* antitumour activity against a variety of solid malignancies and myeloid leukaemia (Kikuchi *et al*, 2001; Nieda *et al*, 2001). Relevant to their practical application in human immune therapies, they can be potently activated by agents such as *α*-galactosylceramide (KRN7000). We chose the U937 lymphoma cell line as the NKT-cell target (Nicol *et al*, 2000; Takahashi *et al*, 2000) because U937 cells express high levels of antiapoptotic Bcl-2 (Lickliter *et al*, 2003). The second model is also relevant to clinical immunotherapy, with the use of A02 human melanoma cells as the target and a Melan-A/MART-1-specific cytotoxic T-lymphocyte (CTL) clone as effector cells. Like U937 cells, A02 cells strongly express Bcl-2 and were therefore suitable for investigating the combined effects of immune attack and exposure to a Bcl-2 inhibitor.

## MATERIALS AND METHODS

### NKT cells

Peripheral blood mononuclear cells (PBMC) were separated from peripheral blood of healthy adult volunteers (*n*=3) by density centifugation over Ficoll-Paque PLUS (Amersham Biosciences, Uppsala, Sweden). Informed consent was obtained from donors before blood collection, and the research was approved by the Human Research Ethics Committees of the Royal Brisbane and Women's Hospital, and Queensland Institute of Medical Research. Following isolation, PBMC were resuspended in AIM-V medium (Gibco BRL, Melbourne, Australia) supplemented with 10% foetal calf serum (Gibco BRL), penicillin/streptomycin, 100 ng ml^−1^ KRN7000 (Kirin Brewery, Japan) and 10 U ml^−1^ interleukin-2 (IL-2; R&D Systems, Sydney, Australia), and incubated at 37°C in a 5% CO_2_ atmosphere. After 7 days, the activated and expanded NKT cells were positively selected using an anti-V*α*24 monoclonal antibody (Beckman Coulter, Sydney, Australia) and magnetic beads (miniMACS system, Miltenyi Biotec, Sydney, Australia). The frequency of NKT cells at specific points during their expansion and purification was assessed with an XL-MCL flow cytometer (Beckman Coulter) after staining with anti-V*α*24-fluorescein isothiocyanate (FITC), anti-V*β*11-phycoerythrin (PE) and anti-CD3-phycocyanin 5 (PC5) antibodies (Beckman Coulter). Results were analysed with EXPO32 software (Beckman Coulter).

### Bcl-2 expression in target cells

The A02 melanoma cell line was derived at the Queensland Institute of Medical Research from a mechanically disaggregated fresh surgical melanoma specimen taken from an HLA-A0201 positive patient. A02 cells and U937 lymphoma cells were grown at 37°C in a 5% CO_2_ atmosphere using RPMI 1640 medium supplemented with 10% foetal calf serum, penicillin and streptomycin. To determine Bcl-2 expression in the U937 cells, aliquots of 5 × 10^5^ cells were fixed and permeabilised using the IntraPrep kit (Immunotech, France) according to the manufacturer's instructions. Cells were then stained with 5 *μ*l of FITC-conjugated anti-Bcl-2 monoclonal antibody (clone 124; Dako, Carpinteria, CA, USA) or an IgG_1_ isotype control (BD Biosciences, San Jose, CA, USA), and examined with an XL-MCL flow cytometer. Results were analysed using Summit version 3.1 software (Cytomation Inc. Fort Collins, CO, USA). Bcl-2 expression in the A02 cells was determined by Western blotting. Cells were resuspended in lysis buffer (50 mM Tris pH 7.6, 250 mM NaCl, 5 mM EDTA pH 8, 50 mM NaF and 50 mM PMSF) and centrifuged. The supernatant was then collected and 100 *μ*g of protein was separated by SDS-polyacrylamide gel electrophoresis and electroblotted onto a PVDF membrane (Bio-Rad). The membrane was incubated with anti-Bcl-2 monoclonal antibody (clone 124, Dako) at a dilution of 1 : 200, followed by a secondary horseradish peroxidase-conjugated rabbit anti-mouse IgG antibody (Dako) at a dilution of 1 : 5000. Binding of antibodies was visualised by enhanced chemiluminescence (ECL; Amersham Biosciences). To confirm even loading of the gel lanes with protein, the membrane was re-probed with an anti-*β*-actin antibody (clone AC-15; Sigma Aldrich, Sydney, Australia).

### Preparation of compounds

The HA14-1 small-molecule inhibitor of Bcl-2 was purchased from Maybridge (Tintagel, UK; product code BTB02933). The compound was dissolved in dimethyl sulphoxide (DMSO), diluted in cell-culture medium to a concentration of 500 *μ*M and stored at −70°C. A fresh aliquot was thawed and used immediately in each experiment. ABT-737 (a gift from Abbott Laboratories, Abbott Park, IL, USA) was dissolved in DMSO and stored at −20°C.

### Cytotoxicity assays

Purified NKT cells were seeded into round bottom 96-well plates at a density of 2 × 10^5^ cells well^−1^ in a final volume of 200 *μ*l of AIM-V medium/10% foetal calf serum. U937 cells were washed in serum-free medium and stained with the membrane dye PKH26 (Sigma), according to the manufacturer's instructions. PKH26-labelled U937 cells were then added to wells containing NKT cells for an effector:target (E : T) ratio of 20 : 1 (donor 1) or 10 : 1 (donors 2 and 3), and coincubated at 37°C in 5% CO_2_ for a total of 4 h. After the initial 1 h of incubation, either 25 *μ*M of the HA14-1 Bcl-2 inhibitor or DMSO vehicle alone was added to the coculture wells and to control wells containing U937 cells alone. To evaluate for apoptosis, cells were washed in annexin–V-binding buffer (10 mM HEPES pH 7.4, 140 mM NaCl and 2.5 mM CaCl_2_), and stained with 5 *μ*l of annexin-V-FITC (BD Biosciences) and 10 *μ*l (0.5 *μ*g) of 7-amino-actinomycin D (7-AAD; BD Pharmingen). A minimum of 10 000 events per sample was then acquired on an XL-MCL flow cytometer and results were analysed using EXPO32 or Summit version 3.1 software. U937-cell viability was defined as the percentage of PKH26^+^ cells that was negative for annexin-V.

The 1H3 antimelanoma CTL clone was derived from PBMC isolated from an HLA-A0201 positive melanoma patient and recognises the Melan-A/MART-1 melanoma antigen. Cryopreserved 1H3 cells were thawed and stimulated at 20 000 cells well^−1^ in a U-bottom 96-well plate containing irradiated (3000 rad) feeder cells (1 × 10^5^ allogenic PBMC and 2 × 10^4^ cells well^−1^ BLCL). Stimulatory medium consisted of RPMI 1640 containing 5% pooled human sera, 100 U ml^−1^ IL-2 and 1 *μ*g ml^−1^ phytohemagglutinin-L (PHA-L). After 7 days, half of the medium volume was replaced with medium containing 5% pooled human sera and 100 U ml^−1^ IL-2 without PHA-L. For all experiments, clones were used at least 7 days after the start of expansion. A02 melanoma cells were de-adhered from tissue culture flasks using trypsin without EDTA and stained with PKH26 dye (Sigma), according to the manufacturer's instructions. The labelled A02 cells were then added to U-bottom 96-well plate containing 1H3 cells to achieve an E : T ratio of 0.2 : 1, and the cocultures were incubated at 37°C in 5% CO_2_ for a total of 4 or 8 h. After the initial 1 h of incubation, either 1 *μ*M of ABT-737 or the corresponding amount of DMSO vehicle alone was added to the coculture wells, and to control wells containing A02 cells alone. Apoptosis was subsequently evaluated by annexin-V and 7-AAD staining (as described above), followed by analysis with a FACSCalibur flow cytometer and CellQuest Pro version 5.2 software (BD Biosciences). At least 10 000 events were acquired per sample.

### Inhibition of perforin/granzyme-B

To investigate the role of the perforin/granzyme-B pathway, immune effector cells were preincubated for 2 h with the perforin inhibitor concanamycin-A (Sigma) at 100 nM (NKT cells) or 1000 nM (1H3 cells), or with the corresponding amount of DMSO vehicle alone (Kataoka *et al*, 1996). The effector cells were then washed twice with phosphate-buffered saline and used in cytotoxicity assays similar to those described above.

## RESULTS

### Expansion and purification of NKT cells

**Figure 1:**
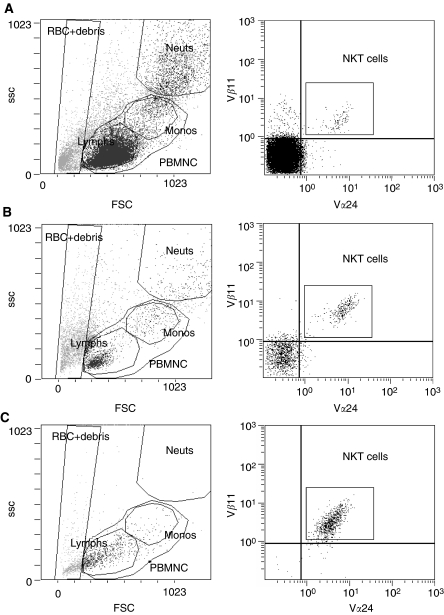
Expansion and purification of NKT cells from normal donor PBMNC. Typical results before expansion (**A**), after 7 days of expansion culture (**B**) and following magnetic separation of V*α*24^+^ cells (**C**) are shown. Cells were stained with anti-V*α*24-FITC, anti-V*β*11-PE and anti-CD3-PC5 antibodies, and then analysed by flow cytometry. Right panels were gated on the PBMNC region defined in the left panels, and V*α*24^+^ V*β*11^+^ NKT cells were counted in the region shown. Abbreviations: FSC, forward scatter; SSC, side scatter; RBC+debris, region-containing erythrocytes and debris; Neuts, neutrophils; Monos, monocytes; Lymphs, lymphocytes.

Freshly isolated PBMC from all normal donors studied contained detectable numbers of NKT cells (Figure 1; Table 1). These numbers markedly increased following 7 days of stimulation with IL-2 and KRN7000, resulting in NKT-cell frequencies of 18–36% of mononuclear cells. Following magnetic separation, populations of V*α*24^+^ V*β*11^+^ NKT cells that were 79–96% pure were obtained. Consistent with an NKT-cell phenotype, these cells were also positive for CD3 (data not shown).

### Cytotoxic effects of combined NKT cells and HA14-1 on U937 lymphoma cells

**Figure 2:**
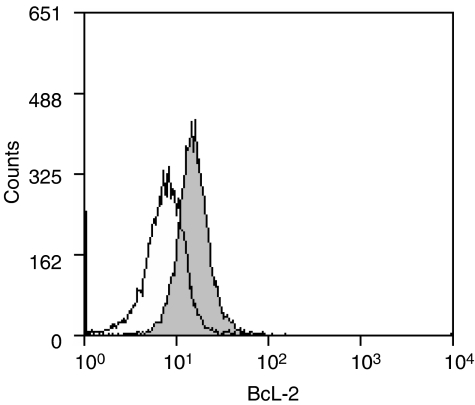
Bcl-2 expression in U937 cells. The histogram plot shows flow-cytometry results after intracellular staining of U937 cells with FITC-conjugated anti-Bcl-2 antibody (gray plot) or isotype control antibody (open plot).

**Figure 3:**
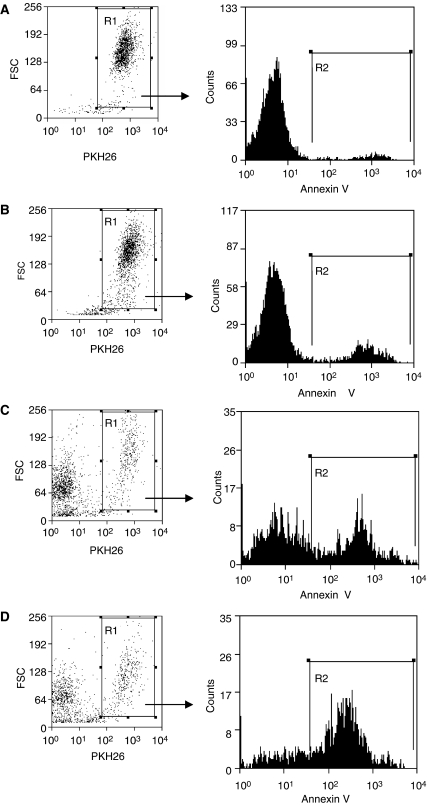
Effect of the HA14-1 Bcl-2 inhibitor on cytotoxicity induced in U937 lymphoma cells by NKT cells. PKH26-labelled U937 cells were coincubated with purified NKT cells at a 10 : 1 effector:target ratio, either with HA14-1 (**D**) or with vehicle alone (**C**). U937 cells incubated without NKT cells were also exposed to HA14-1 (**B**) or vehicle only (**A**). After 4 h, the cells were stained with annexin-V-FITC and 7-AAD, and then analysed by flow cytometry. The proportion of U937 target cells undergoing cell death was determined by first gating on PKH26^+^ cells (region R1, left panels) and then enumerating annexin-V^+^ events (region R2, right panels) in the gated population.

**Figure 4:**
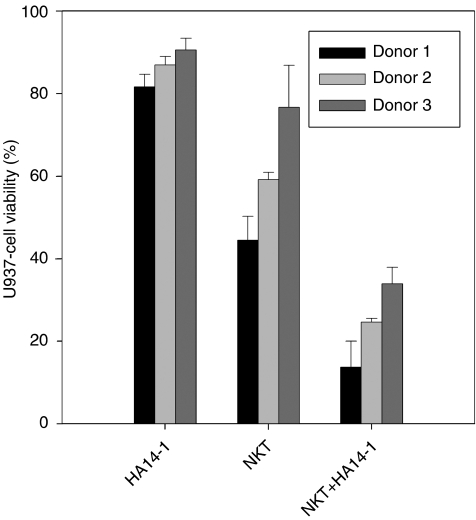
Cytotoxicity of NKT cells from three normal donors in combination with HA14-1, determined as shown in Figure 3. Bars represent mean U937-cell viability in triplicate assays, normalised to the viability of control cells. Results following exposure to HA14-1 alone (HA14-1), NKT cells plus DMSO vehicle (NKT) and NKT cells plus HA14-1 (NKT+HA14-1) are shown. Error bars indicate the s.d.

Bcl-2-expressing U937 lymphoma cells (Figure 2) were coincubated with purified NKT cells from each normal donor. To test the effect of suppressing Bcl-2 function on the susceptibility of U937 cells to NKT-cell cytotoxicity, cocultures were supplemented either with the HA14-1 small-molecule Bcl-2 inhibitor or with vehicle alone. Viability of the U937 target cells was then evaluated with a well-established flow-cytometry technique (Fischer *et al*, 2002; Lin *et al*, 2004), as shown in Figure 3. This involved utilising the PKH26 fluorescent membrane dye to distinguish U937 cells from NKT effector cells and FITC-conjugated annexin-V to stain dying cells. Therefore, viable and dying U937 cells were identified as PKH26^+^/annexin-V^−^ and PKH26^+^/annexin-V^+^ populations, respectively. Figure 3 illustrates that HA14-1 on its own triggered only a low level of cell death at the concentration used in these experiments. Natural killer T cells in the absence of HA14-1 were cytotoxic; however, combined exposure to both NKT cells and the Bcl-2 inhibitor resulted in a significant amplification of cytotoxicity with a corresponding marked increase in annexin-V^+^ U937 cells. As shown in Figure 4, the enhanced cytotoxicity induced by coexposure to HA14-1 was observed in experiments with NKT cells isolated from all three normal donors tested. The mean U937-cell viability in the cocultures decreased from 44.5 to 13.7% (donor 1), 59.1 to 24.6% (donor 2) and 76.6 to 33.9% (donor 3) with the addition of HA14-1. Compared to the effects of NKT cells alone, combination with the Bcl-2 inhibitor therefore reduced target-cell viability by an additional 56–69%. Importantly, since HA14-1 on its own reduced the viability of U937 cells by only 9–18%, our results indicate that coexposure to NKT cells and HA14-1 enhanced target-cell destruction in a synergistic fashion.

**Figure 5:**
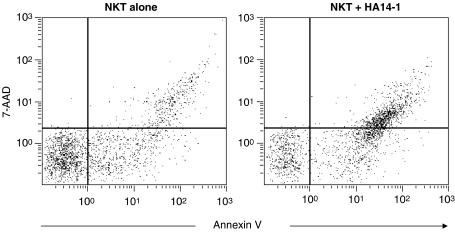
Analysis of 7-AAD staining of U937 target cells. Cocultures of NKT cells and PKH26-labelled U937 cells were stained with annexin-V-FITC and 7-AAD, and then analysed by flow cytometry. Plots gated on PKH26^+^ cells are shown for cocultures supplemented with vehicle only (NKT alone) or with HA14-1 (NKT+HA14-1).

Inhibition of Bcl-2 with HA14-1 should induce apoptosis rather than primary necrosis. We therefore investigated the mode of U937-cell death in the cocultures by analysing the pattern of 7-AAD staining in PKH26^+^/annexin-V^+^ cells. As described previously (Philpott *et al*, 1996), necrotic cells stain brightly for 7-AAD, whereas apoptotic cells become dimly positive. Figure 5 shows results from a typical coculture experiment after gating on the PKH26^+^ population. Exposure to NKT cells in the absence of HA14-1 resulted in annexin-V^+^ U937 cells that were 7-AAD negative, 7-AAD dim and 7-AAD bright. Significantly, coexposure to HA14-1 caused a marked increase in the 7-AAD-dim subpopulation, indicating that the increase in U937-cell death was predominantly owing to apoptosis.

### CTL killing of melanoma cells is amplified by the ABT-737 Bcl-2 inhibitor

**Figure 6:**
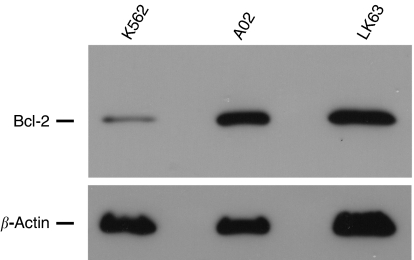
Western blot showing expression of Bcl-2 in A02 melanoma cells compared with that in cells with low expression (K562) and high expression (LK63) levels. The blot was probed with an anti-Bcl-2 monoclonal antibody and subsequently with an anti-*β*-actin antibody, as a loading control.

**Figure 7:**
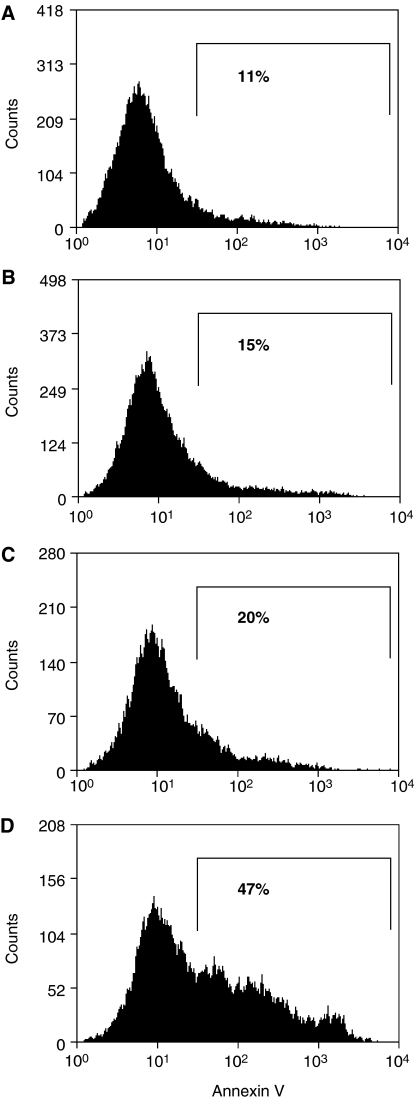
Destruction of A02 melanoma cells by an activated CTL clone is enhanced by coexposure to the small-molecule Bcl-2 inhibitor ABT-737. PKH26-labelled A02 cells were coincubated with the Melan-A/MART-1 specific T-cell clone 1H3 at an E : T ratio of 0.2 : 1. After 1 h, either 1 *μ*MABT-737 (**D**) or vehicle alone (**C**) was added to the cocultures. Control wells with A02 cells alone were also exposed to 1 *μ*MABT-737 (**B**) or vehicle only (**A**). After a total of 4 h, the A02 target cells were evaluated for cell death by staining with annexin-V-FITC and 7-AAD, followed by flow cytometry analysis. The histograms shown were gated on events positive for PKH26 and negative for 7-AAD, to exclude non-target cells and necrotic target cells. The percentages shown below the markers represent the percentage of annexin-V^+^ events and therefore enumerate apoptotic A02 cells.

Figure 6 shows that the A02 melanoma cell line strongly expresses antiapoptotic Bcl-2. The expression level is significantly higher than in K562 cells and is comparable to that in the LK63 acute lymphoblastic leukaemia line, which expresses high levels of the Bcl-2 oncoprotein that influence its survival (Lickliter *et al*, 2003). We investigated whether inhibiting Bcl-2 in A02 cells with the potent small-molecule inhibitor ABT-737 would sensitise them to destruction by a Melan-A/MART-1-specific antimelanoma CTL line (Figure 7). Effector 1H3 cells were coincubated with target A02 cells at a low E : T ratio (0.2 : 1), to better simulate the conditions encountered *in vivo* in clinical immunotherapy. Target cells were again labelled with PKH26 and wells were supplemented with either ABT-737 at 1 *μ*M or vehicle alone after the initial 1 h of coculture. A proportion of the PKH26^+^ target cells (<20%) was positive for 7-AAD; however, this was not significantly different between control wells containing A02 cells alone and coculture wells supplemented with vehicle alone or ABT-737 (data not shown). This population likely represents A02 cells that sustained mechanical damage at baseline when de-adhered from tissue culture flasks. Flow-cytometry analysis plots were therefore gated on PKH26^+^ events that were negative for 7-AAD, so as to exclude this background cell death. The proportion of annexin-V^+^ cells in the gated population (indicated as percentages in Figure 7) is a measure of the specific killing of A02 cells by the CTL clone and/or ABT-737. Typical results from one of the three independent experiments are shown in Figure 7. This indicates that either 1H3 antimelanoma T cells or the ABT-737 Bcl-2 inhibitor caused significant destruction of the A02 cells on their own. In contrast, when A02 cells were exposed to both CTL and ABT-737, there was a substantial amplification of target-cell killing. In the experiment shown, CTL alone triggered a 9% increase in A02 cell death over baseline, whereas CTL and ABT-737 induced a 36% increase. As ABT-737 alone was associated with only a 4% increase in cytotoxicity, the combined treatment resulted in supraadditive destruction of the melanoma cells. Importantly, the increase in A02 cell death was due to an increase in the annexin-V^+^ 7-AAD^−^ subpopulation and therefore specifically to an increase in apoptosis.

### Role of perforin/granzyme-B

**Figure 8:**
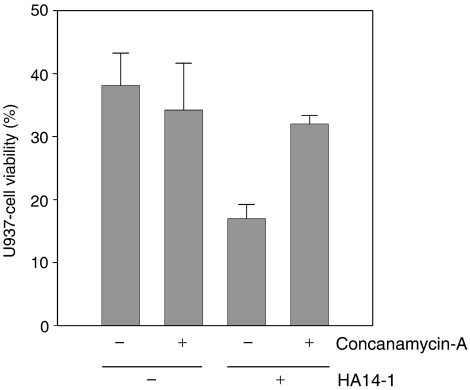
Effect of inhibiting the perforin/granzyme-B pathway. The cytotoxicity of NKT cells preincubated with either concanamycin-A or control medium was assessed in combination with HA14-1 or vehicle only, using the method shown in Figure 3. Bars indicate mean U937-cell viability in triplicate assays and error bars the s.d.

We next investigated whether blocking the perforin/granzyme-B effector pathway of immune cells would affect the enhanced target-cell killing associated with coexposure to a Bcl-2 inhibitor. NKT cells from donor 2 were preincubated with either the perforin inhibitor concanamycin-A or vehicle alone, before establishing cocultures with U937 target cells. Typical results from one of two independent experiments are shown in Figure 8 and reveal that inhibition of perforin did not significantly alter NKT-cell killing of U937 cells in the absence of HA14-1. This suggests that the perforin/granzyme-B system did not significantly contribute to the baseline cytotoxicity of NKT cells from this donor against the U937-cell targets. In contrast, the increase in U937-cell death induced by coexposure to HA14-1 was almost completely blocked by preincubation of the NKT cells with concanamycin-A. These results suggest that the perforin/granzyme-B pathway plays an essential role in the amplification of cytotoxicity against U937 cells mediated by HA14-1, at least in the case of NKT cells isolated from donor 2. We also investigated the role of perforin and granzyme-B in the augmentation of 1H3-cell cytotoxicity against A02 melanoma cells associated with coexposure to the ABT-737 Bcl-2 inhibitor. In this model, inhibition of perforin with concanamycin-A almost completely abolished CTL cytotoxicity, both in the presence and absence of ABT-737 (data not shown). This indicates that 1H3 cells killed their A02 targets almost exclusively via the perforin/granzyme-B pathway. Consequently, the significant increase in killing of A02 cells observed with ABT-737 coexposure potentially reflects a heightened susceptibility to apoptosis induction by granzyme-B.

## DISCUSSION

We investigated the effects of inhibiting the Bcl-2 antiapoptosis oncoprotein on the susceptibility of Bcl-2-overexpressing tumour cells to cytotoxic immune cells. NKT cells derived from three normal donors all showed markedly increased cytotoxicity against Bcl-2-positive U937 lymphoma cells when combined with the HA14-1 small-molecule inhibitor of Bcl-2. In addition, a CTL antimelanoma clone specific for Melan-A/MART-1 revealed significantly more killing of Bcl-2-overexpressing A02 melanoma cells in the presence of the potent Bcl-2 inhibitor ABT-737. Consistent with the cellular physiology of Bcl-2 inhibition, target tumour cells were predominantly induced to undergo apoptosis by combined treatment with immune effector cells and a Bcl-2 inhibitor. Our findings therefore support the hypothesis that Bcl-2 expression in the target U937 and A02 cells suppressed the proapoptotic effects of antitumour immune cells, and that inhibition of Bcl-2 can remove this suppression and allow apoptosis of the tumour cells to proceed.

The perforin/granzyme-B system is an important mechanism of CTL-mediated tumour cell destruction and also contributes to NKT-cell cytotoxicity against U937 cells (Takahashi *et al*, 2000; Voskoboinik and Trapani, 2006). Moreover, Bcl-2 expression can block apoptosis induced by granzyme-B (Pinkoski *et al*, 2001). We therefore investigated whether sensitization to the effects of the perforin/granzyme-B system could explain the increased target cell apoptosis observed with coexposure to a Bcl-2 inhibitor. In the case of NKT cells, pretreatment with the perforin inhibitor concanamycin-A resulted in no reduction in the killing of U937 cells in the absence of HA14-1, but caused almost complete abrogation of HA14-1-mediated cytotoxicity amplification. One explanation for these results is that Bcl-2 overexpression in the U937 cells caused essentially complete suppression of granzyme-B-induced apoptosis. This was reversed by HA14-1 binding to Bcl-2, resulting in increased killing of U937 cells via the perforin/granzyme-B mechanism. As perforin inhibition by concanamycin-A abrogated almost all of this increase, it follows that the perforin/granzyme-B system was the predominant pathway affected by the Bcl-2 inhibitor in the cells tested. In the case of 1H3 T-cells, destruction of A02 melanoma cells was mediated almost exclusively via the perforin/granzyme-B system, either in the presence or absence of the Bcl-2 inhibitor. The marked increase in cytotoxicity associated with coexposure to ABT-737 in this model is therefore likely due to sensitization to the pro-apoptotic effects of perforin/granzyme-B. From a mechanistic standpoint, it is significant that the pro-apoptotic BH3-only protein Bid is cleaved to its active form by granzyme-B, allowing it to trigger cytochrome-*c* release from mitochondria, caspase activation and apoptosis (Ida *et al*, 2003). Bcl-2 expressed by tumour cells could sequester activated Bid (Letai *et al*, 2002), and therefore block apoptosis in the face of immune cell-derived granzyme-B. However, coexposure to BH3 mimetic compounds (such as HA14-1 and ABT-737) could potentially displace activated Bid from Bcl-2, and thus promote cell death.

Our results suggest that combining a Bcl-2 inhibitor with clinical immunotherapy may result in enhanced anticancer efficacy. Moreover, Bcl-2 inhibitors may be especially useful in tumours where the perforin/granzyme-B system is the main functional pathway available for target cell killing. For example, FasL and TRAIL do not effectively signal apoptosis in some tumour cells (Mace *et al*, 2006), and therefore targeting the perforin/granzyme-B mechanism may be essential to immunological antitumour activity in these cases. Effective small-molecule inhibitors of Bcl-2 and related antiapoptosis proteins are now in late preclinical and early clinical development. For example, a phase I study in solid tumours of GX15-07 (a pan-Bcl-2 family inhibitor developed by Gemin X Biotechnologies, Montreal, Canada) has already commenced. The Abbott Laboratories compound ABT-737 has shown considerable promise in preclinical studies, and clinical trials of this (or related) molecules may follow (Oltersdorf *et al*, 2005). Consequently, clinical trials of Bcl-2 inhibitory drugs in combination with anticancer immunotherapy may soon be feasible. Bcl-2 inhibition, however, might also induce apoptosis in activated immune cells, and this could limit its application in immunotherapy strategies involving the *in vivo* activation of antitumour lymphocytes. It is therefore possible that Bcl-2 inhibitors will be most useful in conjunction with adoptive immunotherapy. For example, infusions of immune cells activated by cytokines *ex vivo* could be combined with a Bcl-2 inhibitor in a temporal sequence similar to that used in the current *in vitro* study.

## Figures and Tables

**Table 1:** NKT-cell frequencies in samples from three normal donors, evaluated using the method shown in Figure

	**Day 0**	**Day 7**	**Postseparation**
Donor 1	0.1	36.1	95.7
Donor 2	0.5	17.9	94.5
Donor 3	0.1	18.9	79.0

Results before expansion (Day 0), after 7 days of expansion culture (Day 7) and after magnetic separation of V*α*24^+^ cells (postseparation) are shown, expressed as a percentage of total mononuclear cells.
